# Perceptions of Controlling Teaching Behaviors and the Effects on the Motivation and Behavior of High School Physical Education Students

**DOI:** 10.3390/ijerph15102288

**Published:** 2018-10-18

**Authors:** Juan Antonio Moreno-Murcia, Elisa Huéscar Hernández, Lorena Ruiz

**Affiliations:** 1 Centro de Investigación del Deporte, Universidad Miguel Hernández de Elche, Avda. de la Universidad, s/n, 03130 Elche, Alicante, Spain; 2 Departamento de Psicología de la Salud, Edificio Altamira, Universidad Miguel Hernández de Elche, Avda. Universidad, s/n, 30202 Elche, Alicante, Spain; ehuescar@umh.es (E.H.H.); klera_lorka@hotmail.com (L.R.)

**Keywords:** controlling teaching styles, teachers, physical education, intrinsic motivation, students, adolescents

## Abstract

The purpose of this study was to examine the effects of controlling teacher behavior on high school physical education students’ global intrinsic motivation, perceptions of the importance of the subject matter, intentions to be physically active, level of physical activity, and life satisfaction. The sample in this study was comprised of 416 Spanish high school students. Support for the study’s expectations was provided through structural regression analysis. The analysis revealed that a controlling teaching style was negatively associated with the global intrinsic motivation of the students. In turn, global intrinsic motivation predicted the perceived importance of the subject matter, which explained physical activity intentions. Physical activity intentions were positively associated with level of physical activity, which, in turn, explained life satisfaction. The knowledge obtained in this study can be of benefit to teachers and can be beneficial to the design of more adaptive learning environments for students.

## 1. Introduction

The interpersonal behavior adopted by teachers of physical education classes can have an important influence upon students [1,2,3,4]. The negative effects that may accompany direct instructional styles and pressuring behaviors from teachers can include a lack of interest and involvement in the subject matter by students at later ages [5,6,7]. In the Physical Education context, recent work indicates that a great deal of instructional success depends on the approach that teachers assume in teaching the content, which, in turn, affects student motivation [8,9]. As such, the nature of the classroom instructional climate as shaped by the teaching methods of the instructor is of great relevance to the goal of achieving a positive physical education experience and, in turn, in improving the motivation of students such that they can better engage in positive physical activity experiences over the course of a lifetime.

Self-Determination Theory (SDT) [10] is a contemporary theory that has contributed to our understanding of motivated behavior in social contexts. The theory addresses motivation in terms of an individual’s level of voluntary engagement underlying their behavior. Vallerand’s [11,12] Hierarchical Model of Intrinsic and Extrinsic Motivation (HMIEM) complements Self-Determination Theory. This theory provides an explanation of the role of motivational variables at the situational level, such as the perceived competence of the student in any given class, and subsequent effects at the contextual level, such as their overall perceived ability in physical education. In turn, effects can be felt at the global level, such as the individual’s level of commitment to a physical-activity-related lifestyle. According to the theory, current experiences can have a long-term effect. A third perspective, the Transcontextual Model [13] further attempts to explain how motivation in the school environment can translate to other contexts, such as leisure-time behaviors. With respect to effects at the global level, research provides support for the expectation that physical activity contributes to psychological well-being [14].

In accordance with these theoretical perspectives, the physical education teacher, as a social agent, can exert an important motivational influence through their behavioral interactions with students. The aforementioned patterns of teacher-student interaction can be conceptualized along a behavioral continuum. It ranges from very controlling, such as providing extrinsic incentives and creating a performance- or ego-oriented climate, to the teacher providing an autonomy-supportive environment that is task-oriented and intended to strengthen the intrinsic motivation of the student, but is not necessarily bipolar [15]. Research supports the view that when autonomy support is provided to students, students increase their motivation and commitment, develop stronger intentions to be physically active, and better understand conceptual knowledge such as about developing healthy behavioral practices [16]. This line of research also indicates that increased intrinsic motivation is an outcome that results from autonomy-supportive and task-oriented climates in physical education [17]. Specifically, more self-determined students (as reflected by greater intrinsic motivation) engage in more physical activity and have more favorable attitudes toward the practice of physical activity and sport than do less self-determined students. In addition, these favorable orientations translate into stronger intentions to engage in the subject matter, both in the present as well as in the future [18,19].

Despite the fact that numerous investigations have demonstrated that autonomy support for individual students has beneficial effects on student learning processes [20,21], the tendency for teachers, in general [7], and in physical education specifically [19], is to be more controlling than autonomy-supportive. A teacher-provided control harms students because it frustrates their autonomy, and arouses anger and anxiety, so the instructional behavior has a former cluster with categories related to pressuring language (e.g., “you should” or “now it is time to work”). Overall, controlling teachers make sure students do what they tell them they have to do. As a consequence of the prior discussion and in relation to the need to better understand the effects of controlling teaching styles on adolescent students, this work was designed to examine the extent to which controlling teaching behavior affects global intrinsic motivation, the perceived importance of the subject matter, physical activity intentions, the level of actual physical activity, and life satisfaction in adolescent physical education students. It was anticipated that controlling teaching behaviors would be negatively associated with intrinsic motivation, which would contribute to a lower evaluation of the importance of the subject matter on behalf of students. In turn, lower perceived importance of physical education would be associated with weaker intentions to engage in physical activity, which would result in lower actual physical activity. Actual levels of physical activity involvement would be associated with life satisfaction, with greater involvement predicting higher levels of satisfaction.

## 2. Method

### 2.1. Participants

The sample was comprised of 416 students in their second year of high school who attended seven public educational institutions from the same autonomous community in Spain. The sample included 229 males and 187 females ranging from 16 to 18 years of age (*M* = 16.71 years, *SD* = 0.73 years).

### 2.2. Measures

#### 2.2.1. Controlling Teaching Style

To assess controlling teaching styles, a version of the *Controlling Coach Behavior Scale* (CCBS) developed by Bartholomew, Ntoumanis, and Thøgersen-Ntoumani [22] and modified and validated for the Spanish physical education context by Castillo et al. [23] was used. The modified instrument assesses controlling behaviors of teachers on student learning tasks. The measure contains 15 items which are grouped along four different dimensions including the use of rewards (“My teacher tries to motivate me by promising to reward me if I do well”); negative consequences (“My teacher is less accepting of me if I have disappointed him/her”); use of intimidation (“My teacher shouts at me in front of the others to make me do certain things”); and excessive personal control (“My teacher expects my whole life to center on my physical education participation”). The stem to the questions is, “In reference to my physical education teacher…” and a Likert-type response format is used with response choices ranging from “totally disagree” (1) to “totally agree” (7). Cronbach alpha indices of internal consistency were 0.69, 0.73, 0.85, and 0.78, respectively, for the four subscales. For this study, however, a single score for controlling teaching behavior was used that collapsed the four dimensions, and the Cronbach alpha value of internal reliability of the global scale was 0.94. Confirmatory factor analysis was conducted which initially resulted in a less-than-adequate fit (χ2(94, 416) = 408.26, *p* = 0.00; χ2/df = 4.34; comparative fix index (CFI) = 0.90; normed fit index (NFI) = 0.87; Tucker Lewis index (TLI) = 0.87; standardized root mean square residual (RMSR) = 0.06). Noting that the standardized regression weights and item loadings for questions 3 and 7 were not significant, we thus decided to conduct a second confirmatory analysis without these two items. This analysis resulted in an acceptable fit: χ2(67, 416) = 229.44, *p* = 0.00; χ2/df = 3.42; CFI = 0.94; NFI = 0.92; TLI = 0.92; RMSR = 0.05.

#### 2.2.2. Global Intrinsic Motivation

The variable of intrinsic motivation was assessed through a subscale of the *Behavioral Regulation in Sport Questionnaire* (BRSQ) which was developed by Lonsdale, Hodge, and Rose [24] and modified and validated for the Spanish physical education context by Moreno-Murcia, Marzo, Martínez-Galindo, and Conte [25]. This scale consists of four items with the stem, “When I participate in physical activity and sport”. A sample response is, “because I enjoy it”. The Cronbach alpha internal consistency estimate for this scale was 0.89 and confirmatory factor analysis also revealed adequate fit: χ2(2, 416) = 0.03, *p* = 0.98; χ2/df = 0.19; CFI = 0.99; NFI = 0.99; TLI = 0.99; RMSR = 0.00.

#### 2.2.3. Importance of Physical Education

A three-item scale was used to assess students’ perceptions of the importance and utility value of physical education. The measure was developed by Moreno, González-Cutre, and Ruíz [26] and consists of three items. A sample question was, “I think it is important to take physical education classes”. The response format ranges from “totally disagree” to “totally agree” on a four-point, Likert-type scale. The internal consistency of the scale was 0.76 and confirmatory factor analysis found acceptable fit to the scale: χ2(3, 416) = 0.05, *p* = 0.67; χ2/df = 0.45; CFI = 0.99; NFI = 0.99; TLI = 0.99; RMSR = 0.00.

#### 2.2.4. Physical Activity Intentions

The *Intention to be Physically Active Scale* [27] as adapted to the Spanish language [28] was employed in the present study. The scale is comprised of five items that measure individuals’ intention to be physically active. A sample question is, “After I finish my schooling I would like to maintain my physical activity”. Respondents answer in relation to the stem phrase, “With respect to your intention to practice a physical activity or sport” and the response format conforms to a five-point, Likert-type structure with endpoints of “totally disagree” and “totally agree”. The assessment of internal consistency revealed a Cronbach’s alpha value of 0.81 and confirmatory factor analysis yielded good indices of fit: χ2(5, 416) = 25.77, *p* = 0.00; χ2/df = 5.15; CFI = 0.97; NFI = 0.96; TLI = 0.94; RMSR = 0.04.

#### 2.2.5. Life Satisfaction

The Scale of Life Satisfaction as developed by Vallerand, Blais, Briére, and Pelletier [29] was used in this study. The Spanish language modification of the scale was developed through work by Atienza and colleagues [30,31]. The scale consists of five items grouped on a common factor that utilizes the stem phrase that refers to “Satisfaction with your life…” A sample question is, “My life corresponds with my ideals” and a seven-choice, Likert-type format is used with endpoints of “totally disagree” and “totally agree”. The Cronbach alpha estimate of internal consistency was 0.83 and good fit was obtained through confirmatory factor analysis: χ2(5, 416) = 19.90, *p* = 0.00; χ2/df = 3.98; CFI = 0.98; NFI = 0.97; TLI = 0.96; RMSR = 0.03.

#### 2.2.6. Habitual Physical Activity

The Spanish language version [32] of the Habitual Physical Activity Questionnaire [33] was used to assess actual physical activity behavior in the present study. Free-time physical activity was assessed through four questions. The first question refers to the type of sport or physical activity that the individual engages in and the weekly and monthly frequency in which the physical activity is carried out. A formula is utilized that reflects intensity, time, and type of physical activity that is carried out on a monthly basis [34]. The three remaining questions assess physical activity in one’s free time (e.g., “During my free time, I engage in sport or physical exercise”) and uses a 1–5 scale ranging from “never” to “with great frequency”. An overall value for habitual physical activity is computed for physical activity by computing the mean of the four responses.

### 2.3. Procedure

The directors of the various secondary education schools were contacted to inform them about the purpose of the study and to solicit their involvement. Upon attaining approval from these individuals for the involvement of their students, the next step was to inform the parents of prospective students about the purpose and procedures of the study and to obtain their written consent before students were contacted. Upon receiving parental written consent, the verbal assent of student participants was requested. Student participation was entirely voluntary and the individual questionnaires were completed anonymously. The study has the approval of the ethics committee of the principal investigator institution (DPS.JMM.01.17).

Previously to 416 final participants measurements, 26 were eliminated because some of the questionnaires were incomplete (17) and others (9) were rejected to answer any of the scales. Completion of the questionnaires was conducted in the students’ physical education classes and under the supervision of the principal investigator who addressed any questions or uncertainties that students mentioned. The questionnaires were completed individually and required roughly 20 min to complete.

### 2.4. Data Analysis

Descriptive statistics, including means and standard deviations, were calculated for each of the variables included in the study and simple correlations among each of the variables were also computed. The internal consistency of each scale was assessed using Cronbach’s alpha value. Structural equation modeling was employed through the AMOS 21.0 package on SPSS (IBM Corp. Released 2012. IBM SPSS Statistics for Windows, Version 21.0. Armonk, NY, USA: IBM Corp.).

## 3. Results

### 3.1. Descriptive Statistics

The mean value for students’ perceptions of controlling teaching behavior was 2.77. The mean values for global intrinsic motivation and the perceived importance of physical education were 5.39 and 2.77, respectively. In terms of the intentions to be physically active, the mean for the sample was 3.70, the habitual physical activity level was 9.98, and mean life satisfaction scores were 5.40. The correlational analysis revealed that each of the variables had a positive bivariate correlation with the other variables with the exception of controlling teaching style, which had a significant and negative correlation with general intrinsic motivation. However, some values below 0.300 are not very powerful, as, for example, in the case of the life satisfaction variable (Table 1).

### 3.2. Structural Equation Analysis

**Figure 1:**
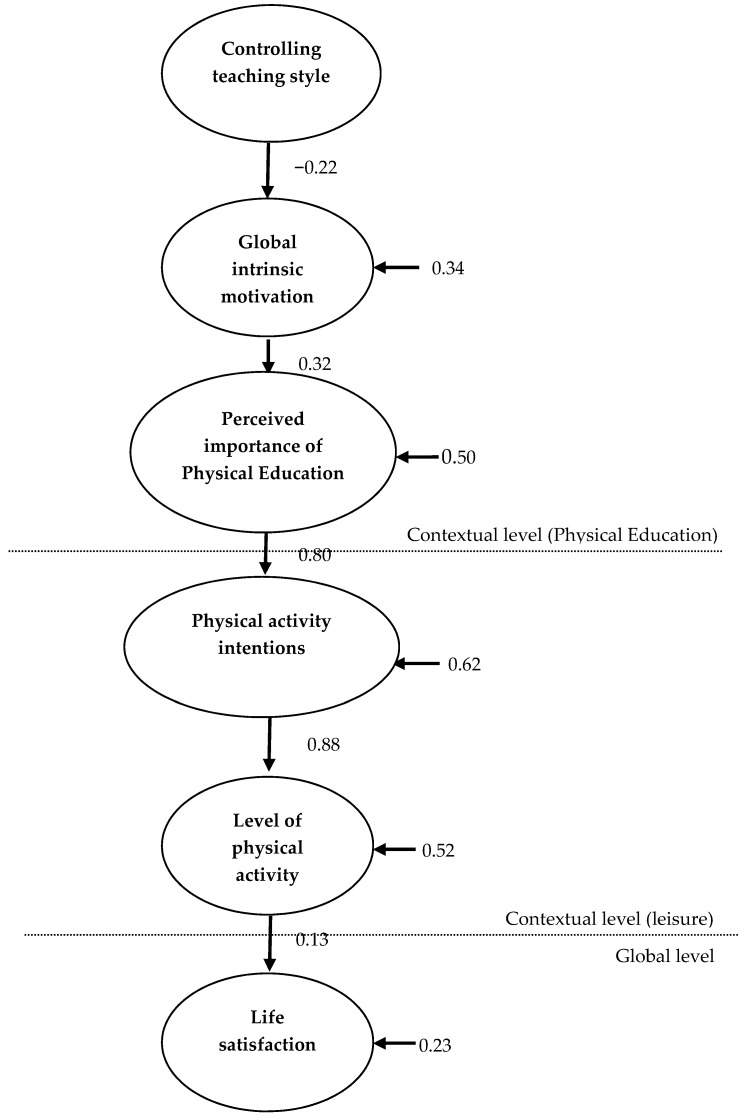
Structural regression model. The parameters are standardized and significant, *p* < 0.05.

Structural equation models were tested, which allowed for the examination of the relationships proposed through the conceptual model. Variables included in the model were controlling teaching style, global intrinsic motivation, perceived importance of physical education, physical activity intentions, level of physical activity, and life satisfaction. As shown in Figure 1, controlling teaching style is represented as an exogenous variable with the remaining variables considered to be endogenous. In this model, controlling teaching styles were negatively associated with global intrinsic motivation, as anticipated. Global intrinsic motivation was positively associated with the perceived importance of physical education which, in turn, explained the intention to be physically active. Intention to be physically active explained level of actual physical activity which, in turn, explained life satisfaction. A maximum likelihood estimation model was employed using the covariance matrix among the items as the starting point for the data analysis. The proposed model achieved an acceptable level of fit, χ2(149, *N* = 416) = 464.68, *p* = 0.00; χ2/df = 3.12; CFI = 0.91; NFI = 0.87; TLI = 0.90; Root mean square error of approximation (RMSEA) = 0.07 with each of the proposed relationships attaining significance. These results indicated that controlling teaching styles were negatively associated with global intrinsic motivation. However, a positive relationship existed between (intrinsic) motivation and life satisfaction as the relationship was influenced by the perceived importance of physical education, intentions to be physically active, and levels of habitual physical activity.

## 4. Discussion

The purpose of this study was to examine the effects of teachers’ controlling behaviors on intrinsic motivation, intentions to be physically active, levels of physical activity engagement, and life satisfaction in adolescent physical education students. The study’s expectations were supported.

In correspondence with previous research in this line of study, the present investigation found that controlling teaching styles were associated with lower intrinsic motivation. Various other studies [1,35,36,37] have also found that students’ perceptions of a controlling teaching style contribute to lower levels of intrinsic motivation which could, in turn, lead to a reduced motive to be physically active as a consequence of negative experiences during physical education classes [38,39].

In agreement with Deci and Ryan’s [40] expectations, a more self-determined motivational profile, as reflected by greater intrinsic motivation, was associated with various positive consequences, including more favorable appraisals of the importance of physical activity, stronger intentions to be physically active in the future, and higher levels of actual physical activity involvement [27,41]. As such, the school environment constitutes an ideal context within which to work to shape sedentary behavioral orientations through more positive student experiences in physical education students [42]. Therefore, favorable physical education experiences can be an important step toward a healthy lifestyle [39]. Thus, in this line, Erturan-liker, Yu, Alemdaroglu, and Kölklü [43] provide relevant information about the role of self-determined motivation in physical education to help improve health.

To date, there have been few studies that have investigated the relationships between the contextual and global levels [44]. In the present study, the level of physical activity (contextual level) predicted life satisfaction (global level) which coincides with the findings reported by previous researchers in the physical activity domain [45]. These results suggest that the design of programs to enhance intrinsic motivation through physical education has an important role in the creation of a supportive motivational climate [9] and can contribute to strengthening the life satisfaction of students. Typical approaches taken within these types of intervention programs include: using informal language that is flexible and not controlling; allowing independent and critical thought; rational discussion with students about the value of respecting the thoughts, feelings, and behaviors of others; striving to reach personal goals; providing interesting and relevant learning opportunities; allowing time for students to work independently; and permitting students to take initiative on their own learning activities [4,6]. Through these teaching behaviors, teachers can foster an autonomy-supportive climate [46,47] and can facilitate a more self-determined motivational approach in their students [48,49].

With regard to the limitations of this study, it is important to note that causality cannot be inferred from the cross-sectional correlational data that was obtained. As such, an experimental design using an actual intervention would be appropriate for examining the effects of variables on each other and over time. Similar studies at different educational levels would also be beneficial, including the use of self-observation and the inclusion of additional variables, such as gender. In sum, this study has used Self-Determination Theory as the primary point of reference for the study of student motivation and takes an important step toward understanding the overall personal development of students.

## 5. Conclusions

Despite these limitations, the present study sheds light on the importance of using adaptative approaches about interpersonal behavior, avoiding pressuring behaviors from controlling teaching styles. Furthermore, this study expands understanding of the use of physical education classes (contextual level) as the ideal context to promote students’ rate of physical activity and increase life satisfaction (global level).

## Figures and Tables

**Table 1:** Descriptive statistics and correlations among variables.

Measures	*M*	*SD*	*α*	1	2	3	4	5	6
1. Controlling teaching style	2.61	1.19	0.94	-	−0.22 *	−0.04	0.04	0.06	−0.07
2. Global intrinsic motivation	5.39	1.45	0.89	-	-	0.43 **	0.66 **	0.48 **	0.16 **
3. Perceived importance of PE	2.77	0.79	0.76	-	-	-	0.53 **	0.33 **	0.13 **
4. Physical activity intention	3.70	0.91	0.81	-	-	-	-	0.66 **	0.22 **
5. Level of physical activity	9.85	5.21	-	-	-	-	-	-	0.17 **
6. Life satisfaction	5.40	1.15	0.83	-	-	-	-	-	-

** *p* < 0.001, * *p* < 0.005; PE = Physical Education.
